# Impact of Tongue Piercings on Oral Health: A Narrative Literature Review

**DOI:** 10.3390/clinpract15090171

**Published:** 2025-09-18

**Authors:** Silvia Rojas-Rueda, Nechama S. Citrin, Mark Adam Antal, Rene Garcia-Contreras, Carlos A. Jurado, Francisco X. Azpiazu-Flores

**Affiliations:** 1 Division of Dental Biomaterials, School of Dentistry, The University of Alabama at Birmingham, Birmingham, AL 35233, USA; 2 Division of Operative Dentistry, Department of General Dentistry, College of Dentistry, The University of Tennessee Health Science Center, Memphis, TN 38104, USA; 3 Department of Operative and Esthetic Dentistry, Faculty of Dentistry, University of Szeged, 6720 Szeged, Hungary; 4 Interdisciplinary Research Laboratory, Nanostructures, and Biomaterials Area, Escuela Nacional de Estudios Superiores (ENES) Unidad Leon, Universidad Nacional Autonoma de Mexico (UNAM), Leon 37684, Guanajuato, Mexico; 5 School of Dentistry, Ponce Health Sciences University, Ponce 00716, Puerto Rico; 6 Division of Restorative and Prosthetic Dentistry, College of Dentistry, The Ohio State University, Columbus, OH 43210, USA

**Keywords:** tongue piercing, oral health, piercing complications, piercing effects, dental trauma, soft tissue injuries

## Abstract

Background: Tongue piercing has gained popularity among teenagers and young adults as a form of self-expression, cultural identity, and fashion. However, patients are often unaware of the harmful effects tongue piercings can have on their oral health. Despite its popularity, this form of body modification carries considerable risk, particularly when performed or maintained without proper care. This review summarizes findings from clinical case reports, observational studies, and previous literature reviews, with a focus on the clinical outcomes of tongue piercings and their appropriate management. Methods: An internet-based literature review was conducted to evaluate the short- and long-term oral health implications of tongue piercings. Only articles published between January 1990 and April 2025 were included. The databases searched were PubMed, Google Scholar, Scopus, and Web of Science, using keywords such as “tongue piercing,” “oral piercing,” “oral complications,” and “dental trauma.” Results: The literature revealed that tongue piercings can lead to numerous adverse effects on oral health, including dental fractures, gingival recession, enamel wear, and localized tissue overgrowth, in addition to localized and systemic infections. The presence of foreign objects in the oral cavity, combined with poor oral hygiene, habitual trauma, and long-term contact with oral tissues, often worsens these complications. Conclusions: The results of this literature review suggest that tongue piercings pose significant and often underestimated risks to oral health. Clinicians should remain vigilant, educate patients on potential complications, and be well-equipped to prevent, monitor, and manage associated dental problems effectively in clinical practice.

## 1. Introduction

Body piercing is defined as the insertion of jewelry into openings made in various parts of the body, including but not limited to the ears, eyebrows, lips, tongue, nose, nipples, and intimate areas [1,2]. This practice dates back to ancient times, where it held cultural and spiritual significance in regions such as Africa, Asia, and South America, often being associated with ritual events, rites of passage, and religious traditions [3,4]. Over the past two decades, body piercing has witnessed a surge in popularity in Western societies, particularly in countries like the United States, the United Kingdom, France, and Germany. Adolescents and young adults form the largest demographic engaging in this practice, making it a rapidly expanding industry with increasing social acceptance and commercial appeal [5,6]. Despite its widespread adoption, the practice of body piercing is carried out in both regulated and unregulated environments. The level of hygiene and sterilization methods employed can vary significantly depending on the location and the practitioner. Many establishments adhere to standardized sterilization protocols, which can increase the risk of complications such as infections and allergic reactions [7,8]. As the trend continues to grow, there is a pressing need for stricter regulations, enhanced public awareness, and improved practices to ensure the safety and well-being of individuals undergoing body piercing.

The literature highlights various intraoral sites for piercing, including the tongue, lips, lingual frenulum, labial frenulum, uvula, and cheek. A summary of the most common areas and negative effects caused by intra-oral piercing can be seen in Table 1. Among these, tongue piercing stands out as one of the most popular forms, particularly among teenagers and young adults. This piercing is typically placed either in the midline or at the tip of the tongue, depending on individual preference [9]. The procedure for tongue piercing is generally performed without local anesthesia. The tongue is protruded, and a hollow needle of the same gauge as the intended barbell jewelry is used to make the perforation. Following the piercing, the jewelry is immediately inserted to maintain the opening [10]. Healing from the procedure typically takes three to five weeks, during which the site requires careful attention to hygiene to prevent complications. After the initial healing phase, the piercing must be worn regularly, as removing the jewelry for extended periods can lead to spontaneous closure of the perforation [11]. Many individuals with tongue piercings also choose to incorporate decorative jewelry, further emphasizing the esthetic appeal of this practice. The motivations behind tongue piercing are varied and often include self-expression, fashion, body art, or making a bold personal statement. For some, it also carries a sense of rebellion or daring, further adding to its allure among younger demographics [12]. While tongue piercings are widely accepted in modern culture, it is important to note the associated risks, such as infections, allergic reactions, and dental complications, underscoring the need for proper care and the professional execution of the procedure.

Tongue piercings have been associated with various negative effects on oral and systemic health as reported in the literature. Common complications include pain, swelling, bleeding, infection, gingival recession, and soft tissue lesions. Additionally, changes in oral microflora, altered salivary composition, compromised periodontal health, and dental damage such as chipped or fractured teeth have also been documented [19]. These adverse effects underline the significant risks associated with tongue piercings. Beyond localized complications, tongue piercings may act as potential vectors for transmitting viruses. The literature has identified a risk of transmission for the human immunodeficiency virus (HIV), hepatitis A (HAV), hepatitis B (HBV), hepatitis C (HCV), herpes simplex virus (HSV), and Epstein–Barr virus (EBV) [20]. This highlights the systemic health implications of tongue piercings, especially in cases where sterilization and hygiene practices are inadequate. A recent survey of 225 young people revealed that up to 53.7% of individuals were not informed about the associated risks before undergoing the procedure [21]. Moreover, tongue piercings have been linked to more specific and severe complications. These include bifid tongue, atypical trigeminal neuralgia, soft tongue tissue lesions, and hypertrophic keloid lesions [22]. Such complications not only affect oral health but can also have an impact on the individual’s overall quality of life, making the need for informed decision-making even more critical. While tongue piercings may serve as a form of self-expression, they pose significant risks that should not be overlooked.

Presently, the existing literature on the complications caused by oral piercings presents limitations related to their research design, heterogeneity and quality of the evidence since much of the evidence is derived from low-quality studies such as case reports and small observational designs, hence limiting the strength and generalizability of their findings. Additionally, systematic reviews frequently include strict inclusion criteria, potentially omitting clinically relevant data. There is also a gap in studies addressing public and professional awareness, with limited attention given to preventive strategies and post-piercing care practices.

This narrative literature review aimed to explore and summarize the impact of tongue piercings on oral health, emphasizing the negative side effects associated with their use. While narrative reviews lack the systematic methodology used in systematic reviews, they offer a broad and adaptable approach to exploring a topic in an emerging field, enabling the synthesis of diverse sources and the identification of trends and gaps in the literature. This flexibility makes narrative reviews especially useful for generating hypotheses and guiding future research directions. This type of review is well known in the field of dentistry, where several respected journals regularly publish high-quality narrative reviews to inform both clinical practice and academic research. In addition to shed light on the potential complications arising from tongue piercings, ranging from localized oral health issues to systemic implications. It underscores the critical importance of informing patients about the risks associated with tongue piercings before placement and advocates for comprehensive patient education. Additionally, this review serves as an educational resource for younger clinicians, equipping them with the knowledge and tools needed to counsel patients effectively on the potential consequences of tongue piercings. By fostering awareness, clinicians can help mitigate adverse outcomes and promote informed decision-making among their patients.

## 2. Materials and Methods

Articles published between January 1990 and March 2025 on the topic of tongue piercings and their impact on oral health were searched on four major medical research databases: PubMed, Google Scholar, Scopus, and Web of Science. The search strategy did not include filters and employed the terms “tongue piercing” and “oral piercing” to identify relevant literature. The initial search yielded a total of 257 articles.

The inclusion and exclusion criteria are outlined in Table 2. Excluded from the review were letters to the editor, books, book chapters, and case reports lacking sufficient detail on the placement, duration, or clinical impact of the piercing. Additionally, studies without full-text availability were omitted. Only peer-reviewed articles that explicitly addressed the oral health consequences of tongue piercings—such as tissue damage, dental trauma, or infections—were included in the final analysis to ensure the reliability and relevance of the findings.

## 3. Results

The literature review conducted revealed multiple publications addressing the oral health effects associated with tongue piercings. To better organize and analyze this information, the selected studies were categorized into two distinct tables based on their type, scope, and clinical focus.

Following a review of the titles and abstracts, 19 manuscripts were selected for inclusion in this review. These were categorized into two main groups: 14 case studies and clinical reports, and 5 literature reviews, each offering an analysis into the oral complications associated with tongue piercings.

Table 3 includes case studies, case reports, and case series, each offering detailed accounts of individual clinical scenarios involving tongue piercings. These publications provide a better understanding of the specific oral health complications such as tooth fractures, gingival recession, soft tissue trauma, mucosal lesions, and both localized and systemic infections. They highlight the diverse ways in which tongue piercings can negatively impact the oral cavity, often influenced by factors such as jewelry type, placement, duration, and oral hygiene. Table 4, on the other hand, compiles literature reviews and meta-analyses that evaluate the clinical outcomes and risks associated with tongue piercings. These sources synthesize data from multiple studies to identify common patterns, summarize complications, and propose evidence-based preventive measures. They also emphasize the importance of patient education, risk assessment, and clinical vigilance.

**Figure 1:**
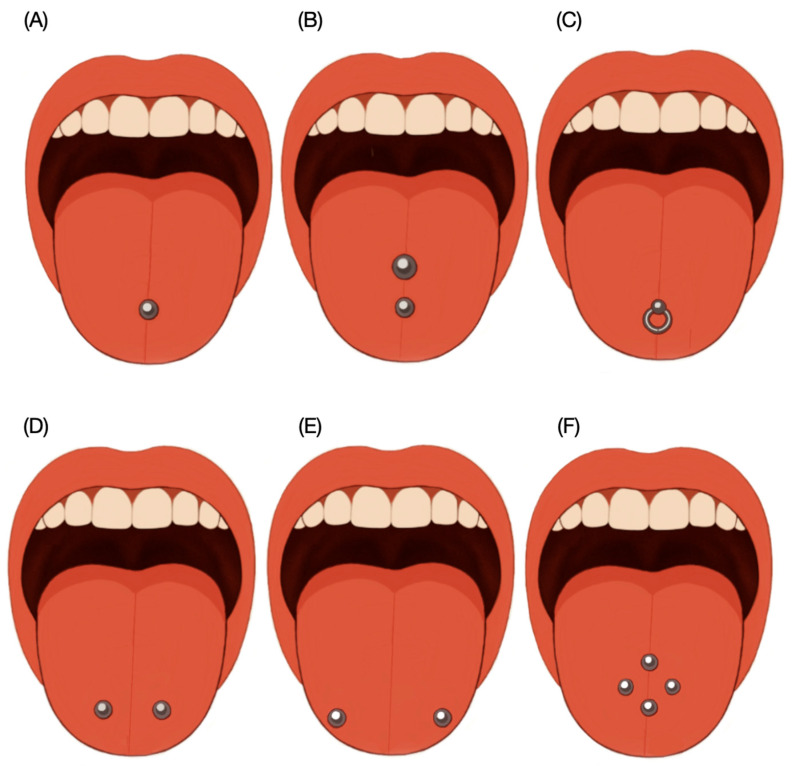
Schematic diagram illustrating the most common sites for tongue piercings. (**A**) Single middle tongue; (**B**) double piercing in midline; (**C**) ring piercing; (**D**,**E**) double piercing horizontally and (**F**) multiple piercing usually involving two or more piercings arranged in a row or symmetrically on either side of the midline.

The reviewed literature identified several common sites for tongue piercings. These include the middle tongue piercing (vertical tongue piercing) with a straight barbell placed at the center of the tongue, and the double tongue piercing, featuring two piercings aligned along the midline. The ring tongue piercing is also common and is generally positioned at the center of the tongue. Additionally, the double lateral tongue piercing consists of two studs placed parallel to each other on either side of the tongue, while the horizontal tongue piercing runs through the tip of the tongue, with ball ends on each side. Finally, there are multiple tongue piercings, which often involve two or more piercings arranged in a row or symmetrically on either side of the midline. A schematic diagram illustrating these piercing sites is provided in Figure 1.

## 4. Discussion

### 4.1. Dental Trauma

Frequent biting or abnormal chewing patterns associated with tongue piercings frequently result in dental trauma, the severity of which can vary depending on the duration of wear and the type of jewelry used. This trauma commonly affects the enamel and dentin and, in more severe cases, may extend to the pulp. Clinical reports have documented fractures of tooth cusps caused by repeated contact between tongue jewelry and the teeth [42,43]. Such damage not only compromises dental function but also has esthetic implications for affected individuals. Tongue piercings have also been linked to specific dental conditions such as cracked tooth syndrome, which presents as cold sensitivity particularly in the lower first molars and is believed to result from tongue jewelry exacerbating pre-existing structural weaknesses [44]. Evidence from the literature highlights that dental damage frequently begins within the first year of wearing a tongue piercing, especially when the original long-shank barbell is not replaced with a shorter one after the recommended two-week healing period [45]. If the jewelry is not replaced, increased mobility and repeated contact with the teeth can heighten the risk of trauma. Additionally, a positive correlation has been found between the duration of piercing use and the severity of dental damage. Prolonged use has been shown to cause cumulative trauma, particularly to posterior teeth, resulting in more extensive damage over time [46].

### 4.2. Gingival and Mucosa Trauma

Clinical reports consistently highlight that tongue piercings can cause significant trauma to oral soft tissues, particularly the gingiva and mucosa. Gingival recession is one of the most frequently documented complications, most often affecting the lingual aspect of the lower central incisors due to repetitive mechanical trauma from the jewelry [47,48,49]. This recession can lead to root exposure, tooth sensitivity, and increased risk of periodontal disease. Studies have shown that up to 80% of individuals with tongue piercings present with measurable gingival breakdown in this area, compared to only 34% in non-pierced control groups [50].

In addition to gingival trauma, tongue piercings can irritate the internal surfaces of the cheeks and palate, often causing redness, swelling, and localized pain [48]. Continuous friction from the jewelry can result in ulceration, mucosal atrophy, and tissue overgrowth [51]. Acute injuries have been observed in up to 50% of individuals with tongue piercings, with lesions sometimes persisting for weeks due to constant movement and exposure to saliva and microbial flora. Chronic irritation may further lead to mucosal edema, reactive fibrotic changes, and localized fibrous hyperplasia, interfering with speech and mastication [52]. Over time, persistent trauma may contribute to submucosal fibrosis or the formation of traumatic fibromas [53].

Other reported complications include erythematous changes in the palatal mucosa indicating localized inflammation, transient alterations in taste perception—possibly due to lingual nerve irritation—and leakage of blood or serum from the piercing site, which may persist beyond the normal healing period and suggest delayed healing or secondary infection [49].

### 4.3. Risk of Hemorrhage

The most commonly reported issues are prolonged bleeding following tongue perforation. Due to the tongue’s dense vascularization, improper technique or incorrect placement can result in damage to blood vessels. If larger vessels are affected, the resulting hemorrhage can be severe and may require urgent medical intervention [54]. In addition to vascular complications, the possibility of nerve damage during the piercing process is a significant concern. Injury to lingual nerves may cause a range of symptoms, from temporary paresthesia to dysesthesia of the tongue, which can affect both speech and oral function. A particularly severe case described in the literature involved sustained bleeding that progressed to hypotensive collapse a life-threatening condition caused by excessive blood loss [55]. This rare but serious event illustrates how tongue piercing, often perceived as a minor cosmetic procedure, can have systemic consequences if complications arise. Such cases highlight the importance of procedural precision, comprehensive anatomical knowledge, and adherence to aseptic protocols. These findings underscore the critical importance of ensuring that tongue piercings are performed by trained professionals with expertise in oral anatomy. Clinicians should educate patients on the potential for serious complications, including bleeding and nerve damage, and emphasize the need for immediate medical attention if such issues occur. Pre-procedural counseling and post-procedural monitoring are essential components of care to minimize risk and ensure patient safety.

### 4.4. Tissue Overgrowth

Tongue piercings subject the oral mucosa to repeated microtrauma, initiating a chronic wound-healing response characterized by collagen deposition and fibrous tissue formation. Experimental histological studies in animal models have demonstrated that initial granulation tissue at the piercing site progressively transforms into dense fibrosis, often accompanied by foreign body granulomas [56]. Clinically, this response presents as fibrous hyperplasia—firm, nodular lesions that may be sessile or pedunculated. These lesions can limit tongue mobility and interfere with essential functions such as speech and mastication. Population-based reviews indicate that soft tissue overgrowth occurs in approximately 16–33% of tongue piercing cases, emphasizing its relevance as a long-term complication [57]. Furthermore, a review of 193 cases of focal fibrous hyperplasia found that over 90% of patients had a history of local trauma or chronic irritation, reinforcing mechanical stress as a major contributor to these proliferative changes [58]. While hyperplastic tissue often regresses after removal of the piercing, persistent or extensive lesions may require surgical excision and histopathological evaluation to rule out other reactive growths or rare neoplasms. In some cases, overgrown tissue has been reported to engulf the jewelry itself, making surgical intervention unavoidable. Histological analysis of these lesions has revealed corrosion particles embedded within multinucleated giant cells, further highlighting the chronic inflammatory and foreign-body response associated with tongue piercings [59].

### 4.5. Localized Infections

Tongue piercings act as direct entry points for microorganisms, making localized infections a common complication. These infections typically present with erythema, edema, pain, and purulent discharge at the piercing site. The tongue’s constant movement, proximity to a diverse microbial population, and challenges in maintaining proper hygiene around the jewelry further increase the risk. Clinical studies report infection rates between 10% and 30% during the immediate post-piercing period [60]. The most frequently identified pathogens include *Streptococcus* spp., *Staphylococcus aureus*, and various anaerobic bacteria that thrive in the oral environment. In some cases, localized infections can escalate to abscess formation requiring incision, drainage, or antibiotic therapy, and in severe instances, progress to life-threatening deep-space infections such as Ludwig’s angina [61]. Risk factors for infection include poor-quality or non-biocompatible jewelry, inadequate sterilization during the piercing procedure, and poor post-procedural hygiene. Piercings placed closer to the posterior region of the tongue may be particularly susceptible due to increased difficulty in cleaning and deeper bacterial colonization. Additionally, behaviors such as smoking, improper aftercare, or sharing utensils can further elevate the risk of infection. Preventive measures include the use of high-grade biocompatible materials such as medical-grade titanium, ensuring piercings are performed by trained professionals, and educating patients on hygiene practices and early signs of infection [62,63].

### 4.6. Systemic Infections

Although less common than local complications, tongue piercings have been linked to systemic infections due to the rich vascular supply of oral tissues, which facilitates the entry of pathogens into the bloodstream. Cases of bacteremia following tongue piercing have been documented and pose a significant risk, particularly for immunocompromised individuals and patients with prosthetic heart valves [64]. Reports of infective endocarditis and septicemia following recent piercings—commonly involving *Streptococcus viridans* or *Staphylococcus aureus*—underscore the seriousness of these complications [60]. Because bacteremia is often transient, diagnosis can be challenging unless systemic symptoms such as fever, malaise, or lymphadenopathy is present. In severe cases, hospitalization and intravenous antibiotic therapy may be necessary [65]. Prophylactic antibiotic coverage has been suggested for high-risk patients undergoing oral piercings; however, recommendations vary across international guidelines [66]. The risk is further elevated with self-administered or non-professional piercings performed under non-sterile conditions and without appropriate post-procedural care. Medical screening prior to piercing should be considered for individuals with underlying health conditions. The potential for systemic dissemination of oral pathogens highlights a broader need for public health education and tighter regulation of body piercing practices [67].

### 4.7. Ingested Piercing

Although most ingested foreign bodies pass through the gastrointestinal tract without complication, the accidental ingestion of tongue piercing components poses a documented health risk. Detachment often occurs unknowingly during eating or sleeping, making early detection difficult. Ingested parts may cause trauma to the oropharynx or, in rare but serious cases, result in gastric retention, appendicitis, or intestinal obstruction [68]. Several case reports have documented the need for surgical retrieval of tongue bars from the appendix or ileocecal region, emphasizing the importance of prompt identification and management [69]. Diagnostic imaging such as abdominal radiographs or CT scans is often necessary to locate the object [70]. If spontaneous passage fails or complications develop, endoscopic or surgical removal may be required [71]. Delayed diagnosis can increase the risk of gastrointestinal perforation, peritonitis, or long-term digestive issues. Risk factors include worn or poorly threaded jewelry, improper design, and lack of patient awareness. Preventive strategies involve the use of secure, medical-grade materials, regular inspection of the jewelry, and prompt medical or dental evaluation if detachment is suspected [72].

### 4.8. Limitations of the Review

This narrative review was not conducted following a predefined and structured protocol for literature search and selection, as is typically required for systematic reviews. Instead, the methodology was more flexible, aiming to provide a broad overview of the available literature on the oral health effects of tongue piercing. The literature search was restricted to English-language publications indexed in four major databases: PubMed, Google Scholar, Scopus, and Web of Science. Although no filters were applied during the search process to maximize literature inclusion, certain types of publications were excluded—specifically, non-indexed studies, conference abstracts, letters to the editor, and articles published in languages other than English. These exclusions inherently limited the scope of the review and may have resulted in the omission of potentially relevant findings. Additionally, no formal risk of bias assessment or quality grading of the included studies was performed, which are typically part of systematic or meta-analytical approaches. The authors acknowledge these limitations may limit the reach of the present review. Additionally, future investigations on this topic with defined inclusion and exclusion criteria, standardized data extraction, and critical appraisal of studies quality, and statistical analysis of the data gathered is required. This would allow for a more rigorous and quantitative evaluation of the clinical effects of tongue piercings and support evidence-based recommendations for dental professionals and patients alike.

### 4.9. Recommendations

Tongue piercings, though popular among adolescents and young adults, pose significant risks to oral and overall health. Complications can include pain, swelling, gum recession, tooth fractures, enamel wear, speech and chewing difficulties, and infections. In rare cases, serious conditions like nerve damage or infective endocarditis may occur. We strongly advise patients to reconsider this type of oral piercing. For patients who still choose to proceed with a tongue piercing, it is essential they are fully informed of these potential risks.

Clinicians should take the time to educate patients about proper oral hygiene, the importance of regular dental checkups, and the need to monitor for early signs of complications. In addition, dental providers must be prepared to diagnose and manage emergencies related to oral piercings, including infections, bleeding, allergic reactions, and soft or hard tissue injuries. Ultimately, prevention through patient education remains the best approach. We strongly encourage patients to reconsider oral piercings in favor of safer alternatives, and we urge clinicians to maintain a proactive stance in counseling and care related to body modifications in the oral cavity.

## 5. Conclusions

Dentists should be aware that tongue piercing remains a popular trend among teenagers and young adults, and they must be prepared to manage the various oral health complications associated with this trend. These may include damage to hard and soft tissues, such as tooth fractures, gingival recession, and soft tissue trauma, as well as localized or systemic infections. Some patients may schedule appointments to seek advice before getting a tongue piercing, and clinicians should be ready to provide clear, evidence-based information regarding potential risks and long-term consequences. Patient education should emphasize the importance of oral hygiene, regular checkups, and early signs of complications to help minimize adverse outcomes.

## Figures and Tables

**Table 1:** Areas of piercing and negative effects reported intra-orally [13,14,15,16,17,18].

Areas Intra-Orally	Negative Effects
Midline of the tongue	Tooth damage
Tip of the tongue	Gingival inflammation
Lower LIP	Lip inflammation
Cheek intra and extra-orally	Cheek inflammation
Lingual frenulum	
Maxillary frenulum	
Uvula	

**Table 2:** Inclusion and exclusion criteria of the search performed.

Criterion	Inclusion	Exclusion
Time period	Publications available between January 1990 and March 2025	All publications published before January 1990
Language	English	Non-English
Type of articles	All research types including primary research (e.g., case studies, in vitro studies, in vivo studies and reviews). Full text available.	Letters, books, book chapters, case reports lacking details on the effect of piercing. No full text not available

**Table 3:** Summary of clinical case reports about tongue piercing [23,24,25,26,27,28,29,30,31,32,33,34,35,36].

Authors/Year	Methods/Clinical Report	Results
Farah et al. (1998) [23]	A 25-year-old Caucasian female sought a dental consultation about getting a tongue piercing. The practitioner advised against it, explaining the potential complications. Despite this, the patient proceeded with the piercing. One week later, she returned with tongue swelling and reported difficulty speaking and chewing. She was prescribed an analgesic mouthwash and oral pain medication.	Dentists should also be able to provide consultation to patients contemplating oral piercing. While many oral piercings probably resolve uneventfully, the wide range of possible adverse outcomes associated with the procedure make it difficult to condone.
Brennan et al. (2006) [24]	An 18-year-old Caucasian female patient presented to the dental emergency department complaining of generalized sensitivity to cold drinks and when breathing. Clinical examination revealed dentin fracture on the palatal aspects of 15 and 24 and lingual of 34 and 36. Patient received a tongue piercing (18 mm barbell shaped). Tooth lesions were restored with resin composite.	With the growing popularity of oral piercing, patients will need to be more informed of potential complications associated with this procedure. Clinicians need to be aware of the potential etiology of dental fractures, secondary to the placement of intraoral jewelry.
Shinohara et al. (2007) [25]	A 16-year-old white male presented to the emergency department with healed mucosa over a tongue piercing placed four months earlier. One month after the piercing, he noticed the sphere had penetrated the central surface of his tongue. Clinical examination revealed a firm, 5 mm mass embedded in the midline. Under local anesthesia, the piercing was surgically exposed, removed, and the site was sutured.	This case reports an oral piercing with part of the hardware embedded beneath the ventral tongue mucosa, likely caused by the patient’s habit of biting and pulling on the dorsal ball before healing. The issue was resolved through surgical removal, and the patient chose not to replace the piercing.
Zadik et al. (2007) [26]	An 18.5-year-old female presented to the dental emergency service with mobility of her lower front teeth. Clinical exam revealed a 2.5 cm metal/plastic tongue piercing on the mid-dorsum, with a bent metal bar and calculus around the plastic sphere near the floor of the mouth. The patient had received the piercing 4.5 years earlier. Lingual gingival recession, 7 mm attachment loss, 4 mm probing depth, and type II mobility were noted in the mandibular incisors. Although informed of the damage caused by the piercing, the patient refused removal and opted to replace it with a shorter one.	Dentists should carefully exam the oral tissue of patients with oral piercing for early diagnosis of these complications. Dental surgeons have the responsibility to educate their patients about these conditions and to recommend appropriate treatment to them.
Berenguer et al. (2006) [27]	A 28-year-old woman presented to the department of periodontology with the chief complaint of “loose hurting teeth” in the lower anterior area. The patient had worn 2 lingual hoops and a mandibular labrette in the form of a bar for the previous 12 years. Clinical evaluation revealed severe periodontitis in the lower anterior teeth, a very unusual condition in a healthy young adult. All the mandibular anterior teeth presented from moderate to severe mobility. Probing depths ranged from 3 to 7 mm. Patient was informed that the cause was the tongue piercing and she decided to discontinue its use.	Dentists and physicians have an ethical mandate to educate patients about potential complications resulting from intraoral jewelry use. While short-term case reports have documented numerous dental injuries related to intraoral piercing, this long-term case report illustrates the potential for such devices to result in rapid periodontal destruction, tooth loss, and eventually loss of normal function.
Correa et al. (2014) [28]	A 23-year-old female presented with pain in the mandibular anterior region. Her medical history was unremarkable, with no tobacco use. Intraoral examination revealed a double-ended metal tongue piercing placed 7 years prior. The piercing had caused a diastema between the mandibular central incisors, with probing depths of 6–8 mm, bleeding on probing, and grade II tooth mobility. Treatment included removal of the piercing and scaling and root planing of the affected sites.	The use of oral piercings has become a fashionable practice worldwide. The mean prevalence of oral and peri-oral piercing, in general, population is 5.2%. It is generally recommended that following healing, such ornaments should be removed daily and cleaned to avoid plaque and calculus accumulation. However, some patients rarely remove their ornaments for cleaning.
Bajkin et al. (2014) [29]	A 15.5-year-old female presented with pain and mild swelling near the lower central incisors. Examination revealed a midline barbell-shaped tongue piercing. Both central incisors were non-vital, mobile, and showed clinical attachment loss. Incisal chipping was noted on both maxillary and mandibular central incisors. The piercing was removed, and the patient received endodontic treatment along with bone grafting for the mandibular incisors.	Bearing in mind that oral piercing is becoming a common practice, oral health professionals should educate the patients and inform them about possible oral health complications associated with this form of body art. It is especially necessary to warn patients with oral piercings about bad habits that could lead to traumatic injuries of teeth and adjacent structures.
Albeshri et al. (2024) [30]	A 27-year-old Hispanic female patient to the periodontics clinic. Patient was referred from a general dentist that removed her tongue piercing after 12 months of use. Piercing created swelling and suppuration in the mandibular anterior region. Probing depths ranged from 6 to 11 mm. Treatment included full mouth debridement and splint in the lower anterior region followed by bone grafting.	Tongue piercing has negative consequences for periodontal health. Correct diagnosis and treatment planning are needed for the management of various periodontal diseases. The presented case was treated successfully via regenerative therapy with a combination of allograft and membrane. The end result was that questionable teeth were saved and restored to periodontal health.
Kretchmer et al. (2002) [31]	A 22-year-old-male presented to the periodontology clinic for evaluation of the mandibular anterior sextant. Periapical and clinical examination revealed localized horizontal bone loss associated with lower central incisors with 6 mm probing depths, plaque and calculus. Tx included prophylaxis, flap reflection to remove supra and subgingival calculus. Patient stopped using the piercing.	The authors of this report remain confident that with the removal of the tongue stud and the stabilization of the bony lesion, the localized inflammation will resolve, and these teeth will be maintained for a significant amount of time.
Shacham et al. (2003) [32]	Piercing has become so popular during the last 20 to 30 years that many physicians are now treating patients with piercings and dealing with its side effects. Authors present 3 cases that illustrate the complications of tongue piercing (i.e., infection, bleeding, and embedded ornaments). Authors describe the methods for inserting the ornaments to illustrate the possible adverse effects.	When a patient does present with an inflamed tongue caused by piercing, the physician should remove the jewelry, perform a local debridement, institute antibiotic therapy, and give the patient chlorhexidine mouthwash. The patient should be closely observed to monitor the spread of infection. The opening through the tongue will spontaneously occlude.
Lopez-Jornet et al. (2005) [33]	A 28-year-old male patient presented with lingual piercing. A week after the piercing placement presented with pain. Two months after the ball became partially buried within the tongue. Treatment was to surgically remove the piercing under local anesthesia. Tongue healed with no further problems.	The type of piercing generally used in the tongue consists of a stud with 2 balls that are screwed to each end. It is inserted in the central, thickest area, always avoiding the lingual frenum, as well as taking care not to damage the vascular nerves. Complications are sufficiently frequent to put into question the safety of piercing, the dangers of which can be considered.
Fleming et al. (2005) [34]	A 17-year-old male referred to the maxillofacial clinic. Patient had a tongue ornament placed one year previously during a period of severe psychiatric disturbances. The area became infected and healed over leaving the tongue divided at the anterior or midline. The tongue was repaired under general anesthetic as a day case. The tongue healed without complication.	Unusual malformations may be attributable to tongue piercings. These abnormalities may occasionally present when ornaments are no longer in situ. Patients contemplating tongue jewelryshould be counseled on early and late complications. Likewise, dentists must be aware of the pitfalls of orofacial jewelry.
Patussi et al. (2014) [35]	A 23-year-old woman was referred with a painless midline nodule on the dorsal tongue, approximately 15 mm in diameter. She had worn a tongue piercing in the same area for 3 years, removed 2 years prior. The lesion was excised, and histopathology revealed an ulcerated lesion with endothelial proliferation and edematous stroma. The patient had a good outcome with no recurrence after 12 months of follow-up.	Oral piercings can cause mechanical trauma, tooth fractures, speech issues, pain, aspiration, lip inflammation, tissue overgrowth, infections, edema, allergies, tongue lacerations, black tongue, galvanism, scarring, increased saliva, interference with X-rays, nerve damage, and paresthesia.
Özdemir et al. (2018) [36]	AA 16-year-old female presented with severe pain and a lesion on the underside of her tongue and floor of the mouth, two months after receiving a barbell-shaped tongue piercing. Examination revealed perforation of the mouth floor. The piercing was removed under lidocaine spray in the operating room, and the patient was prescribed chlorhexidine rinses and analgesics. One week later, the lesion had healed, and the patient reported no further symptoms.	Piercings should be performed by specialists under sterile conditions. Good oral hygiene is essential to prevent bacterial colonization and infection around the piercing site. The tongue jewelry must fit snugly to avoid excessive movement but not so tight as to cause tissue necrosis.

**Table 4:** Summary reviews about tongue piercing [37,38,39,40,41].

Authors/Year	Methods	Results
Hennequin-Hoenderdos et al. (2016) [37]	The research resulted in 1865 papers and after screening by title and abstract 33 papers were selected for full-text reading, of which 17 we excluded because did not match the eligibility criteria. Finally, 15 papers were selected and processed for data extraction.	A significant relative risk was revealed between tongue piercings and an increased incidence of enamel fissures, enamel fractures and gingival recessions (especially int he lingual region of the mandibular incisors). Both lip and tongue piercings were highly associated with gingival recession
Plastargias et al. (2014) [38]	The purpose of this paper is to review the potential complications caused by oral piercings as they are analyzed in the literature. This manuscript also suggests some ways of improving the oral hygiene of the people who wear piercings, and it suggests some methods of ameliorating the negative consequences of piercings.	Oral piercing is not harmless at all. In fact, the vast majority of the reviews and case reports that have been published concerning this issue have agreed that oral piercings pose both a hazardous direct and indirect risk to the soft and hard oral and perioral tissues and they may even pose life-threatening risks.
Theodossy et al. (2014) [39]	A review of the possible complications of tongue piercing is included in the manuscript. A variety of potential complications of oral piercing have been suggested. The most common of which are pain and swelling. Edema of the tongue is a feature of all tongue piercing, because of the vascularity of the area, and can lead to airway compromise as a direct consequence or due to aspiration of the jewelry.	Dentists should be aware of the increasing number of patients with pierced intraoral and peri-oral sites and be prepared to address dental issues, such as potential damage to the teeth and gingiva and risk of oral infection, which may arise as a result of piercing. Dentists also need to provide appropriate guidance to patients who are contemplating body piercing involving oral sites.
Maheu-Robert et al. (2007) [40]	It is a brief review of the current literature on potential complications and adverse consequences of tongue and lip piercings. The objective is to provide a general overview of possible problems that may be encountered by dentists. In addition, authors highlight the urgent need for dentists and doctors to inform target patients of the risks associated with oral piercings.	Tongue and lip piercings represent a significant risk for direct and indirect damage to soft and hard oral tissues. Although much less prevalent, lethal systemic infections may also occur. Considering the growing popularity of intraoral and perioral piercings, dental professionals should be aware of the potential complications associated with this practice and be able to identify those at high risk for adverse outcomes.
Ziebolz et al. (2009) [41]	Dental professionals are seeing an increasing number of patients with oral piercings and as a result they should be able to inform their patients about possible risks and complications associated with such piercings.	The three cases presented here demonstrate some adverse effects of tongue piercings. The most commonly described oral complication is the damage of teeth and the periodontium. Tongue piercing is a personal decision, but it is important patients are fully aware of possible oral health hazards.

## Data Availability

Data are contained within the article.
